# UBE2J1 knockdown promotes cell apoptosis in endometrial cancer via regulating PI3K/AKT and MDM2/p53 signaling

**DOI:** 10.1515/med-2022-0567

**Published:** 2023-02-24

**Authors:** Ping Zhang, Huiping Guo, Fang Zhao, Ke Jia, Fei Yang, Xiaoli Liu

**Affiliations:** Department of Gynaecology, The First People’s Hospital of Zhangjiagang Affiliated to Suzhou University, No. 68, West Jiyang Road, Zhangjiagang 215600, Jiangsu, China; Department of Gynaecology, The First People’s Hospital of Zhangjiagang Affiliated to Suzhou University, Zhangjiagang 215600, Jiangsu, China

**Keywords:** cell apoptosis, endometrial cancer, MDM2/p53 pathway, PI3K/AKT pathway, UBE2J1

## Abstract

Emerging evidence has demonstrated that ubiquitin conjugating enzyme E2 J1 (UBE2J1) exerts pivotal function in many cancers. UBE2J1 was reported to be dysregulated in endometrial cancer (EC). This study was designed to further investigate the regulatory character and mechanism of UBE2J1 in EC. Bioinformatic tools and databases were used to analyze gene expression pattern and gene expression correlation in EC tissues, and the prognosis of EC patients. Gene expression was evaluated by reverse-transcription quantitative polymerase chain reaction. Western blot was used for protein level detection. *In vitro* cell apoptosis was detected by flow cytometry analyses and TUNEL assays. *In vivo* cell apoptosis was evaluated by detecting Bax and Bcl-2 expression in tumor tissues via immunohistochemical and western blot analyses. In this study, UBE2J1 knockdown promoted cell apoptosis in EC cells and in mouse models of EC. PI3K and AKT expression is positively correlated with UBE2J1 level and is related to poor prognosis of EC patients. UBE2J1 knockdown repressed the PI3K/AKT pathway both *in vitro* and *in vivo*. UBE2J1 downregulation decreased MDM2 expression, but increased p53 expression. MDM2 overexpression reverses the promotion of UBE2J1 knockdown on cell apoptosis in EC. Overall, UBE2J1 knockdown induces cell apoptosis in EC by inactivating the PI3K/AKT signaling and suppressing the MDM2/p53 signaling.

## 1 Introduction

Endometrial cancer (EC) is one prevalent malignant tumor of the reproductive system for females in the world, and its incidence is increasing fast in recent years [1]. The potential causes of such increase in global incidence are the epidemic of obesity and the resulting hyperinsulinemia [2–4]. Studies have shown that the majority of patients with EC have promising therapeutic results after standard therapies, and the development of novel drugs for the treatment of advanced EC is encouraging [5,6]. However, the diagnosis and prognosis of advanced-staged EC patients are still poor. Therefore, revealing the mechanisms of EC to develop targeted therapies are vital for improving endometrial outcome.

Ubiquitin conjugating enzyme E2 J1 (UBE2J1) has been reported to exert biological functions on several cancers. For example, UBE2J1 is suggested to be aberrantly expressed in prostatic cancer and its upregulation is closely related to unfavorable prognosis of patients [7]. In addition, the ectopic expression of UBE2J1 in hepatocellular carcinoma (HCC) was reported to induce poor survival in patients with HCC [8]. Importantly, it has been reported that UBE2J1 overexpression promotes EC cell growth, adhesion, invasion, migration as well as epithelial–mesenchymal transition (EMT) through modulating the PI3K/AKT pathway [9]. Our study further explored the biological effects of UBE2J1 on apoptosis of EC cells.

Emerging evidence has revealed that the PI3K/AKT signaling is implicated in the regulatory process of many cancers. For example, activating the PI3K/AKT pathway was suggested to facilitate of ovarian cancer onset and progression [10]. The PI3K/AKT signaling is activated by the interaction between MALAT1 and miR-26a to enhance the malignant phenotypes of colorectal cancer [11]. Additionally, PI3K/AKT signaling has been validated to be implicated in the regulation of EC progression [9].

MDM2/p53 pathway was also suggested to play a pivotal role in the progression of many cancers. MDM2 was found to be abnormally expressed in the p53 pathway in colorectal cancer, which is putatively associated with cell motions and tumor progression [12]. HAX1 reinforces the malignancy of non-small cell lung cancer by the MDM2/p53 pathway [13]. miR-219a-2-3p restrains pituitary adenoma cell proliferation and facilitates cell apoptosis via interacting with the MDM2/p53 pathway [14]. However, only a few reports refer to the role of MDM2/p53 in EC, and a more specific investigation is needed.

This study aimed to explore the role of UBE2J1 in EC cell apoptosis and the underlying mechanism. We hypothesized that UBE2J1 suppresses EC cell apoptosis via regulating the PI3K/AKT and MDM2/p53 pathways. This study may provide more favorable outcomes for the patients suffering from EC.

## 2 Materials and methods

### 2.1 Cell lines

Ishikawa and MFE-296 cells were bought from ATCC. Both cells were cultivated in the dulbecco’s modified eagle medium (Gibco, USA) containing 10% fetal bovine serum in a damp atmosphere with 5% CO_2_ at 37℃.

### 2.2 Cell transfection

To silence UBE2J1, Ishikawa and MFE-296 cells were transfected with short hairpin RNA (sh-RNA) targeting UBE2J1 (sh-UBE2J1) or the corresponding negative control sh-NC (GenePharma, Shanghai, China). To overexpress MDM2, Ishikawa and MFE-296 cells were transfected with pcDNA3.1/MDM2, with pcDNA3.1 as a negative control. The above-mentioned vectors were stably transfected into EC cells for 48 h using Lipofectamine 3000 (Invitrogen). The transfection efficiency was evaluated by reverse-transcription quantitative polymerase chain reaction (RT-qPCR) after 48 h.

### 2.3 RT-qPCR

Total RNA was extracted from EC cells using TRIzol reagent (Takara, Tokyo, Japan). Total RNA was reverse transcribed into first-strand cDNA using a cDNA Reverse Transcription Kit (Takara). A SYBR^®^ Premix Ex TaqTM II reagent kit (Takara) was applied to perform RT-qPCR with an ABI7500 real-time qPCR system (ABI Company, Oyster Bay, NY, USA). Glyceraldehyde-3-phosphate dehydrogenase (GAPDH) served as the internal reference. The relative gene expression was analyzed applying the 2^−ΔΔCt^ method [15]. Sequences of primers used for RT-qPCR are:

UBE2J1: Forward 5′−GTACATCGTACGGACTCCAG−3′,

Reverse 5′−TCATGGAGGTATTCTTAGCTACAG−3′,

GAPDH: Forward 5′−AGAAACGGCTACCACATCCA−3′,

Reverse 5′−CACCAGACTTGCCCTCCA−3′.

### 2.4 Western blot

Cell lysates were collected by radio immunoprecipitation assay buffer with protease inhibitors. Then, 20 μg of protein sample was separated with 10% sodium dodecyl sulfate polyacrylamide gel electrophoresis and transferred onto a polyvinylidene fluoride membrane (Sigma-Aldrich, St Louis, MO, USA). Next, the membrane was blocked with 5% skimmed milk powder, and then incubated overnight at 4°C with the primary antibodies: anti-Bcl2 (ab182858, 1:2,000; Abcam, Shanghai, China), anti-Bax (ab32503, 1:1,000; Abcam), anti-MDM2 (ab259265, 1:1,000; Abcam), anti-p53 (ab90363, 1:250; Abcam), anti-PI3K (ab191606, 1:1,000; Abcam), anti-p-PI3K (ab182651, 1:1,000; Abcam), anti-AKT (ab8805, 1:500; Abcam), anti-p-AKT (ab38449, 1:1,000; Abcam), and anti-GAPDH (ab9485, 1:2,500, Abcam). After being rinsed with phosphate buffer saline (PBS) thrice, the membrane was incubated with secondary antibody at 37℃ for 1 h. The protein bands were visualized using Imaging Analysis System (Odyssey Infrared, USA).

### 2.5 TUNEL assay

TUNEL assay was performed to monitor cell apoptosis using In Situ Cell Death Detection Kit (Roche, Basel, Switzerland). Biologic coloring agent of 4′,6-diamidino-2-phenylindole (Haoran Biotechnology, Shanghai, China) or Merge (Haoran Biotechnology) was used to dye Ishikawa and MFE-296 cells. An EVOS FL microscope (Thermo Fisher Scientific, Waltham, MA, USA) was used to count the positively stained cells.

### 2.6 Flow cytometry

Cell apoptosis was analyzed by flow cytometry. Cells were collected and rinsed with precooled PBS thrice. After being resuspended with 0.3 mL PBS and fixed with 0.7 mL absolute ethyl alcohol, the cells were stored at −20°C for 24 h and centrifuged at 1,000 rpm for 15 min. Next, the cells were washed with PBS twice after the fixation solution was wiped off. Cells were added with 120 μL RnaseA (200 μg/mL), and cultured at 37°C for 30 min. Next, the cells were stained with annexin V-FITC (BD Biosciences, San Jose, CA, USA) and propidium iodide staining solution (Beyotime). At last, cell apoptosis was evaluated with flow cytometry (BD Biosciences).

### 2.7 Tumor xenografts in nude mice

ShRNA targeting UBE2J1 (sh-UBE2J1) and NC shRNA (sh-NC) were synthesized by GenePharma. The sh-UBE2J1 sequences were 5′−GTGAAGAGTCCGGCTGTTA−3′ and 5′−TAACAGCCGGACTCTTCAC−3′. Sh-UBE2J1 and sh-NC were inserted into the pLKO.1 plasmid and then transfected into 293 T cells in parallel with psPAX2 packaging plasmid and pMD2.G envelope plasmid. The 293 T cells were cultured for 48 h at room temperature with 5% CO_2_, and the lentiviruses stably expressing sh-UBE2J1 or sh-NC were harvested and used to infect Ishikawa cells.

BALB/c mice (females, 4–6 weeks old) were bought from Vital River Laboratory Animal Technology Co., Ltd (Beijing, China) and housed under specific pathogen-free condition. Ishikawa cells (4 × 10^6^) with stable sh-UBE2J1 or sh-NC transfection were subcutaneously injected into each mouse. At 28 days post-cell injection, the mice were euthanized by cervical dislocation, and the tumors were excised for further experiments. The animal experiments were approved by the Animal Ethics Committee of The First People’s Hospital of Zhangjiagang Affiliated to Suzhou University (Approved Number: 2021-012; Jiangsu, China).

### 2.8 Immunohistochemistry

Immunohistochemistry was used to detect the expression of UBE2J1, Bcl-2, and Bax in tumor tissues. Briefly, the sections were dewaxed with xylene and rehydrated and then incubated in 3% H_2_O_2_ at room temperature for 15 min to quench endogenous peroxidase activity. Sections were blocked with 5% bovine serum albumin at 37°C for 20 min and then incubated overnight at 4°C with the following primary antibodies: anti-UBE2J1 (1:150, #MA5-26025, Invitrogen), anti-Bax (1:100, #33-6600, Invitrogen), and anti-Bcl-2 (1:200, #MA5-15668, Invitrogen). After washing with PBS, the sections were incubated with a biotinylated secondary antibody (1:1,000, #31430, Invitrogen) for 30 min at room temperature. Tissue sections were then stained with diaminobenzidine, counterstained with hematoxylin, dehydrated, and mounted. The sections were analyzed using a microscope.

### 2.9 Statistical analysis

SPSS 22.0 (IBM Corporation, Armonk, NY, USA) and GraphPad prism 5.0 (GraphPad Software Inc., San Diego, CA, USA) were used to analyze the data. Experimental assays were conducted in triplicate and the data are expressed as mean ± standard deviation. Comparisons were made by student’s *t*-test or one-way analysis of variance followed by Tukey’s *post hoc* analysis. The value of *p* < 0.05 was of statistical significance. The detailed statistics are shown in Table A1.

## 3 Results

### 3.1 UBE2J1 knockdown promotes cell apoptosis in EC

**Figure 1:**
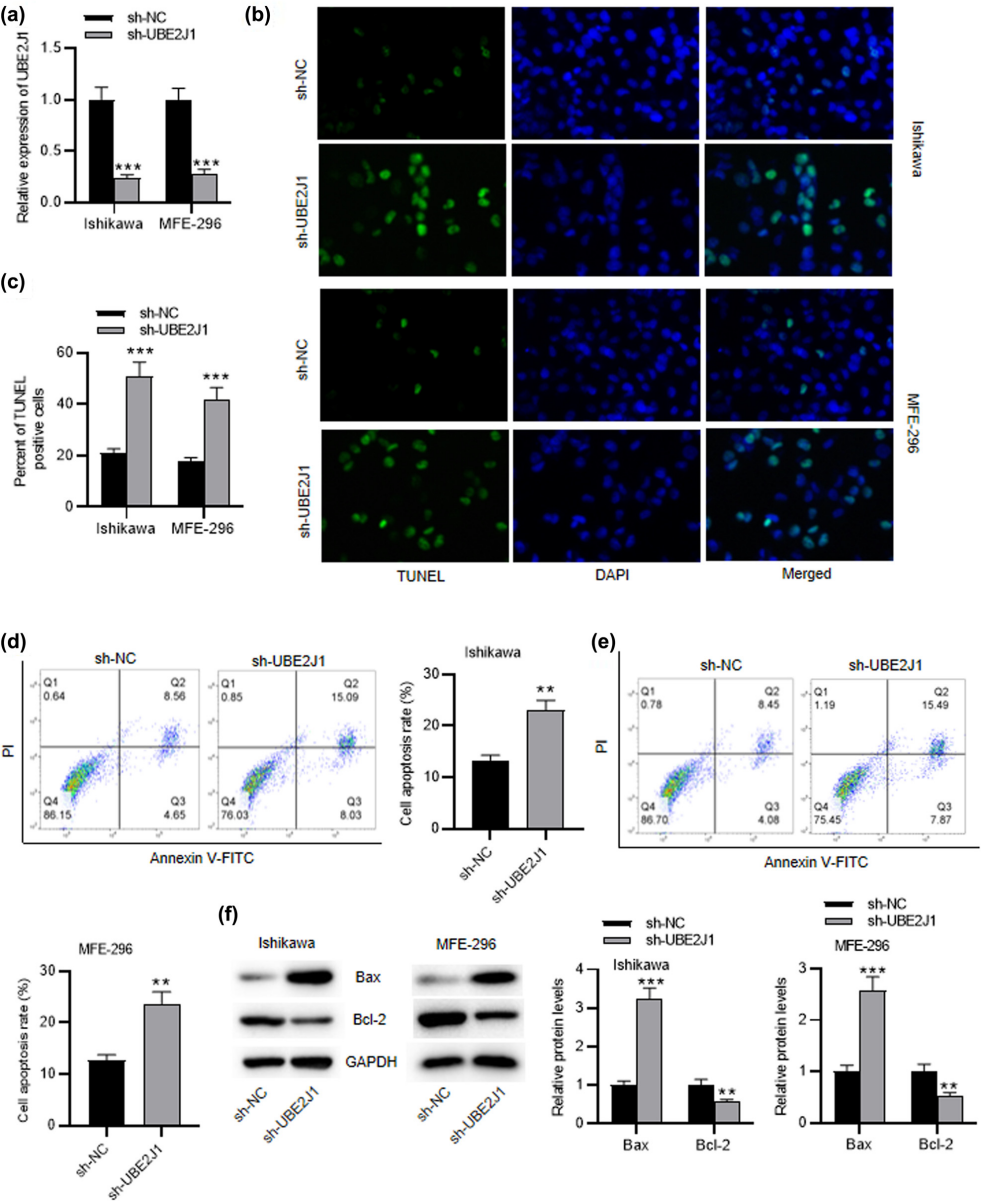
UBE2J1 knockdown promotes cell apoptosis in EC. Ishikawa and MFE-296 cells were transfected with sh-NC or sh-UBE2J1. (a) Expression of UBE2J1 in Ishikawa and MFE-296 cells was evaluated by RT-qPCR. (b and c) TUNEL assay and (d and e) flow cytometry analysis were adopted to measure cell apoptosis. (f) Protein levels of apoptosis markers, Bcl-2, and Bax, in EC cells were analyzed by western blot. Bar graphs were used to represent the data. ^**^ *p* < 0.01, ^***^ *p* < 0.001.

To investigate the role of UBE2J1 in EC cell apoptosis, Ishikawa and MFE-296 cells were transfected with sh-UBE2J1. RT-qPCR showed that the expression of UBE2J1 in EC cells was significantly downregulated after sh-UBE2J1 transfection (Figure 1a). TUNEL assay demonstrated that the percent of TUNEL positive cells was remarkably elevated by UBE2J1 knockdown in Ishikawa and MFE-296 cells (Figure 1b and c). As shown by flow cytometry analysis, the apoptosis rates of EC cells were significantly raised after UBE2J1 knockdown (Figure 1d and e). Subsequently, western blot was conducted to detect the protein levels of cell apoptosis markers Bcl-2 and Bax. The protein level of Bax was markedly increased, while the protein level of Bcl-2 was decreased by UBE2J1 downregulation (Figure 1f). In conclusion, the downregulation of UBE2J1 facilitates EC cell apoptosis.

### 3.2 The expression of PI3K and AKT is positively correlated with UBE2J1 expression and is related to poor prognosis of patients with EC

**Figure 2:**
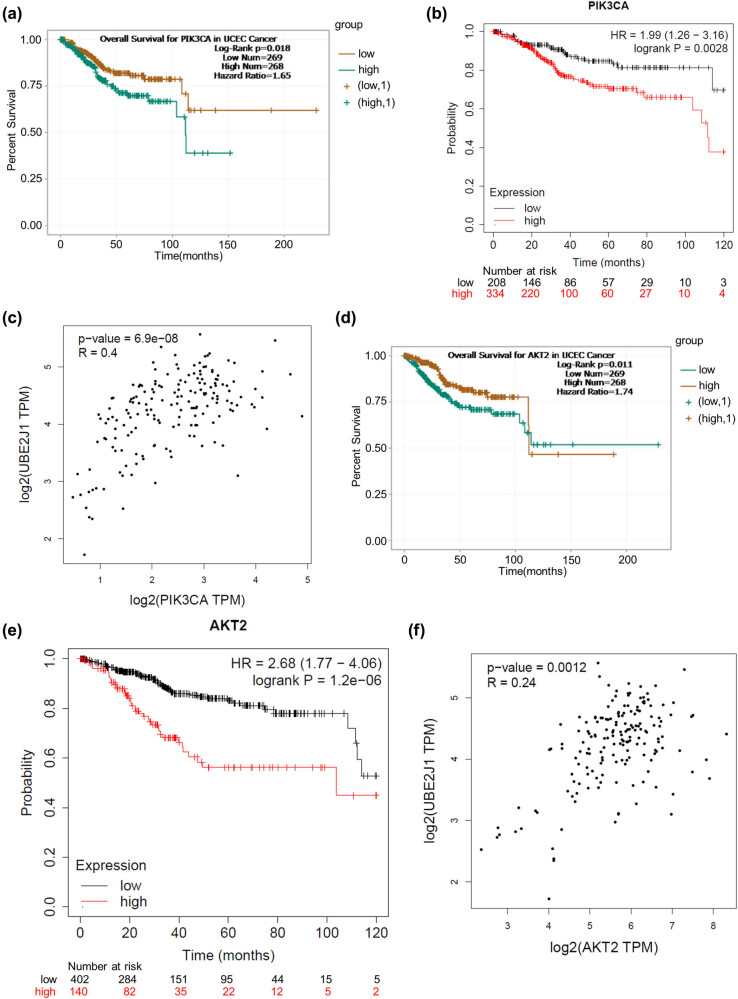
Expression of PI3K and AKT is positively correlated with UBE2J1 expression and is associated with poor prognosis of patients with EC. (a and b) ENCORI (http://starbase.sysu.edu.cn) and Kaplan–Meier plotter websites (http://www.kmplot.com/) show the overall survival time of UCEC patients with high or low PIK3CA expression. (c) GEPIA database (http://gepia.cancer-pku.cn) shows the correlation between UBE2J1 expression and PIK3CA expression in UCEC tissues. (d and e) ENCORI and Kaplan–Meier plotter websites show the overall survival time of UCEC patients with high or low AKT2 expression. (f) GEPIA website shows the relationship between UBE2J1 expression and AKT2 expression in UCEC tissues.

The ENCORI website (http://starbase.sysu.edu.cn) and Kaplan–Meier plotter website (http://www.kmplot.com/) show that high PIK3CA (PI3K) expression is associated with adverse prognosis of patients with Uterine Corpus Endometrial Carcinoma (UCEC) (Figure 2a and b). In addition, GEPIA database (http://gepia.cancer-pku.cn) displays that the expression of PIK3CA is positively correlated with that of UBE2J1 in UCEC tissues (Figure 2c). Moreover, the ENCORI website and Kaplan–Meier plotter website show that high expression levels of AKT2 have close association with the poor overall survival and unfavorable prognosis in patients with UCEC (Figure 2d and e). Furthermore, the GEPIA database displays a positive correlation between UBE2J1 expression level and AKT2 expression level in UCEC tissues (Figure 2f). These results demonstrate that the upregulation of PI3K and AKT is closely related to poor prognosis of patients with EC, and UBE2J1 expression has a positive correlation with PI3K and AKT expression.

### 3.3 UBE2J1 downregulation inhibits the PI3K/AKT signaling pathway in EC cells

**Figure 3:**
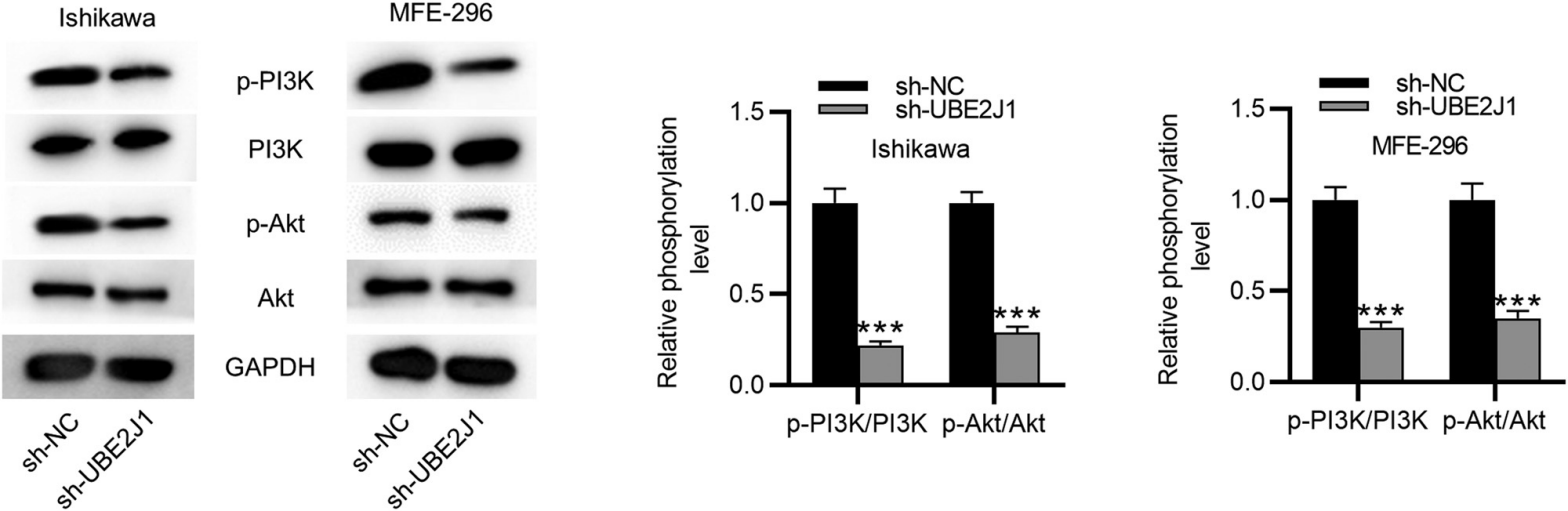
UBE2J1 downregulation inhibits the PI3K/AKT signaling pathway. Western blot analyses were used to detect the protein levels of key molecules involved in the PI3K/AKT signaling pathway in EC cells after downregulating UBE2J1. Bar graphs were used to represent the data. ^***^ *p* < 0.001.

Next, we evaluated the effects of UBE2J1 knockdown on the expression of key molecules involved in the PI3K/AKT signaling pathway in EC cells. The results of western blot showed that UBE2J1 knockdown decreased the protein levels of p-PI3K and p-AKT in EC cells (Figure 3). Therefore, UBE2J1 knockdown inactivates the PI3K/AKT signaling pathway in EC cells.

### 3.4 UBE2J1 knockdown reduces MDM2 expression and facilitates the p53 signaling pathway in EC cells

**Figure 4:**
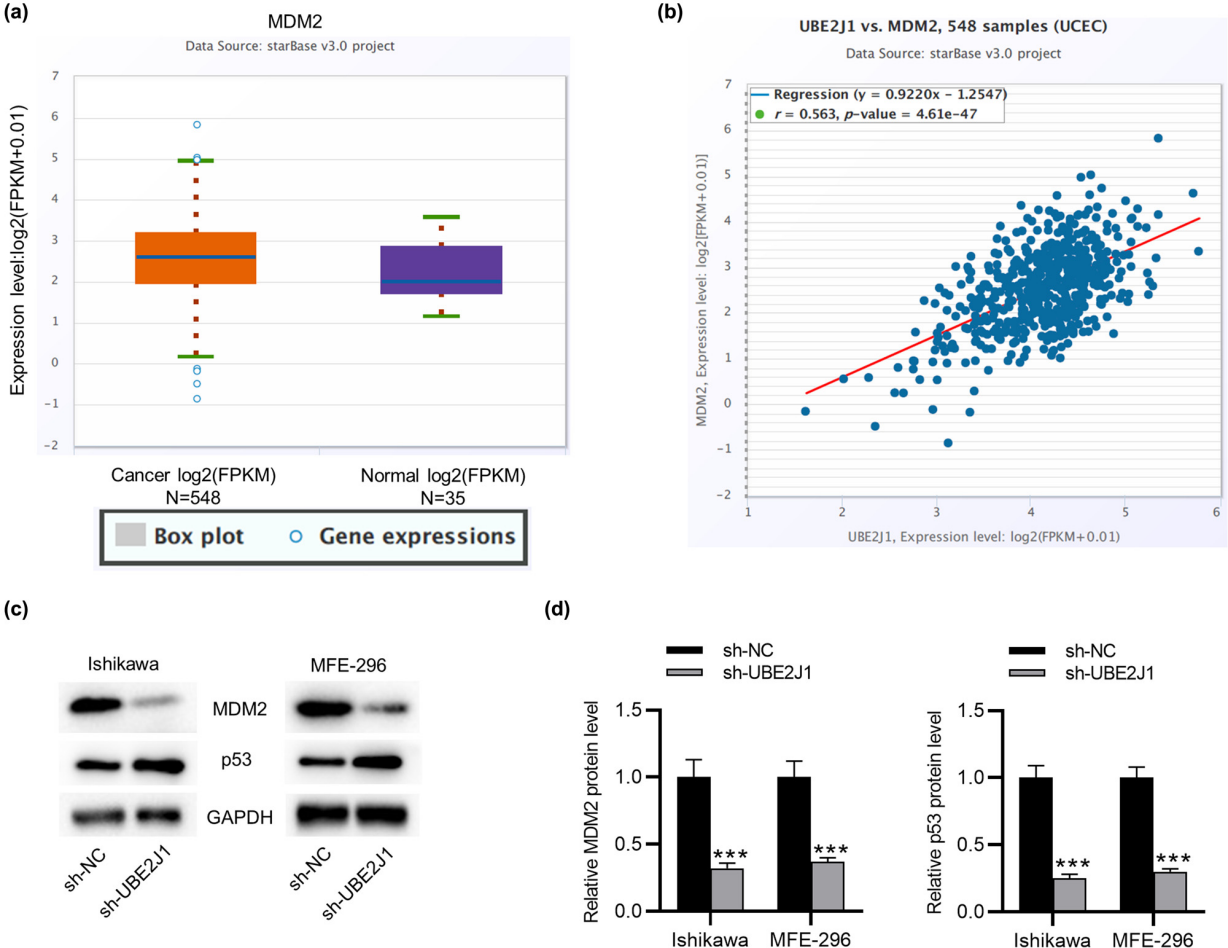
UBE2J1 positively modulates the expression of MDM2. (a) MDM2 expression in 548 EC tissues and 35 normal samples is shown on ENCORI website. (b) ENCORI website shows the correlation between MDM2 expression and UBE2J1 expression. (c and d) Western blot analyses were conducted to measure the protein levels of MDM2 and p53 in EC cells transfected with sh-UBE2J1. Bar graphs were used to represent the data. ^***^ *p* < 0.001.

As shown by the ENCORI website, MDM2 expression is markedly upregulated in 548 UCEC tissue samples versus 35 in normal tissue samples (Figure 4a). Notably, the ENCORI website shows that MDM2 expression is positively correlated with UBE2J1 expression in UCEC tissue samples, indicating that UBE2J1 may influence UCEC development by modulating the expression of MDM2 (Figure 4b). Further, western blot was used to investigate the influence of UBE2J1 knockdown on MDM2 and p53 expression, which revealed that MDM2 protein levels in Ishikawa and MFE-296 cell lines were significantly decreased by UBE2J1 downregulation, while p53 protein levels were increased (Figure 4c and d). The results above indicate that UBE2J1 knockdown promotes the p53 signaling by inhibiting MDM2 expression in EC cells.

### 3.5 MDM2 overexpression reverses the promotive effect of UBE2J1 knockdown on cell apoptosis in EC

**Figure 5:**
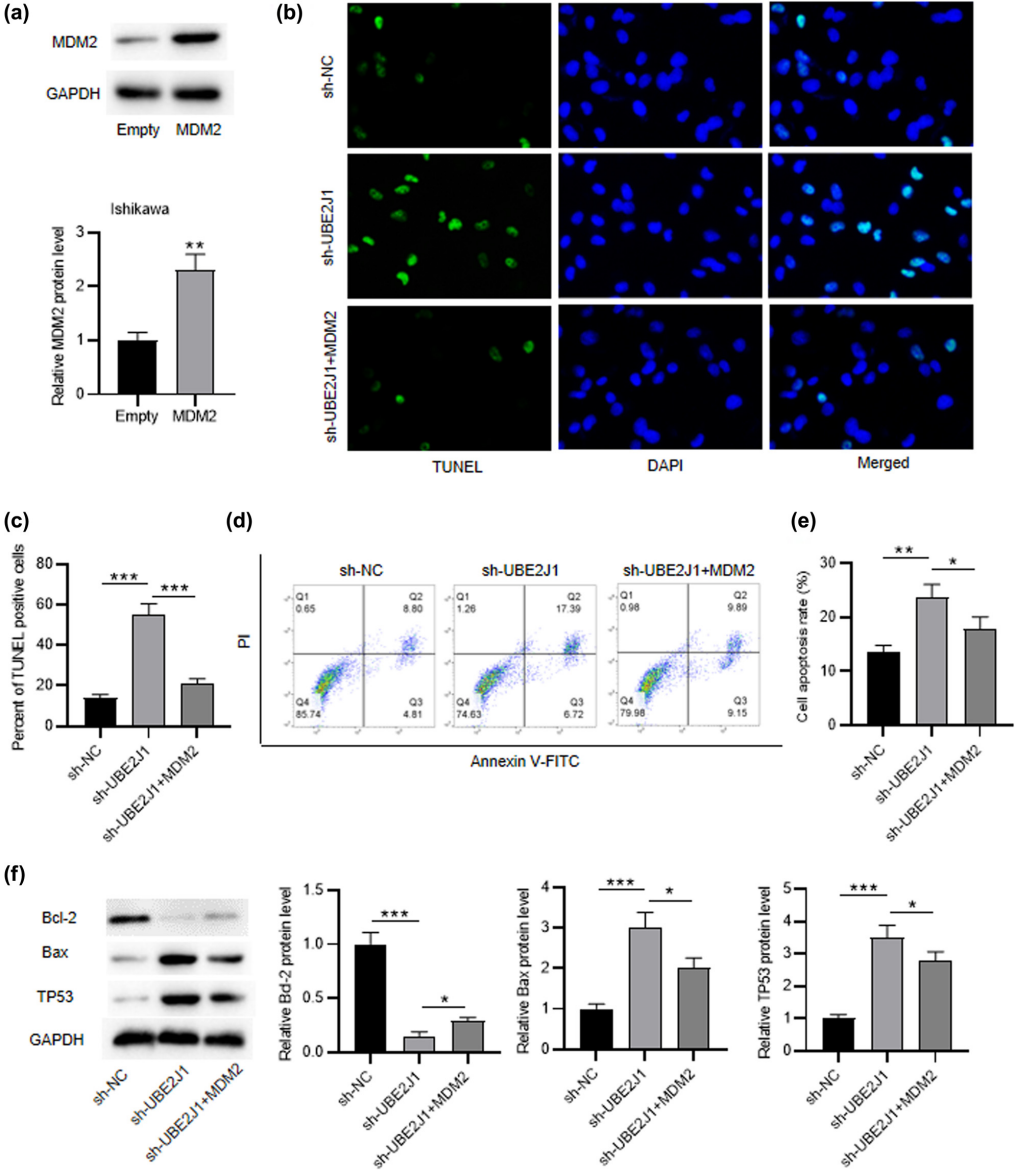
MDM2 overexpression reverses cell apoptosis mediated by UBE2J1 downregulation. (a) Overexpression efficiency of pcDNA3.1/MDM2 in Ishikawa cells was assessed by western blot. (b and c) TUNEL assay and (d and e) flow cytometry analysis were conducted to evaluate the apoptosis of Ishikawa cells transfected with sh-NC, sh-UBE2J1, and sh-UBE2J1 + pcDNA3.1/MDM2. (f) Protein levels of Bcl-2, Bax, and TP53 in Ishikawa cells with the above transfection were analyzed by western blot. Bar graphs were used to represent the data. ^*^ *p* < 0.05, ^**^ *p* < 0.01, ^***^ *p* < 0.001.

To explore the function of MDM2 in modulating EC development, Ishikawa cells were transfected with pcDNA3.1/MDM2. Western blot illustrated that MDM2 expression in Ishikawa cells was markedly increased after overexpressing MDM2 (Figure 5a). Subsequently, TUNEL assay showed that UBE2J1 downregulation increased the percent of TUNEL positive cells, but the promotive effect of UBE2J1 knockdown on cell apoptosis was reversed by MDM2 overexpression (Figure 5b and c). Additionally, flow cytometry analysis showed that the promoting effect of sh-UBE2J1 on the apoptosis rate of Ishikawa cells was reversed by MDM2 upregulation (Figure 5d and e). Moreover, the suppression of UBE2J1 knockdown on Bcl-2 protein level was rescued by MDM2 overexpression, and the promotion of UBE2J1 knockdown on Bax protein level was also neutralized by MDM2 overexpression (Figure 5f). In summary, these results demonstrate that MDM2 upregulation reverses the enhanced cell apoptosis caused by UBE2J1 downregulation.

### 3.6 Knockdown of UBE2J1 induces cell apoptosis in the mouse model of EC

**Figure 6:**
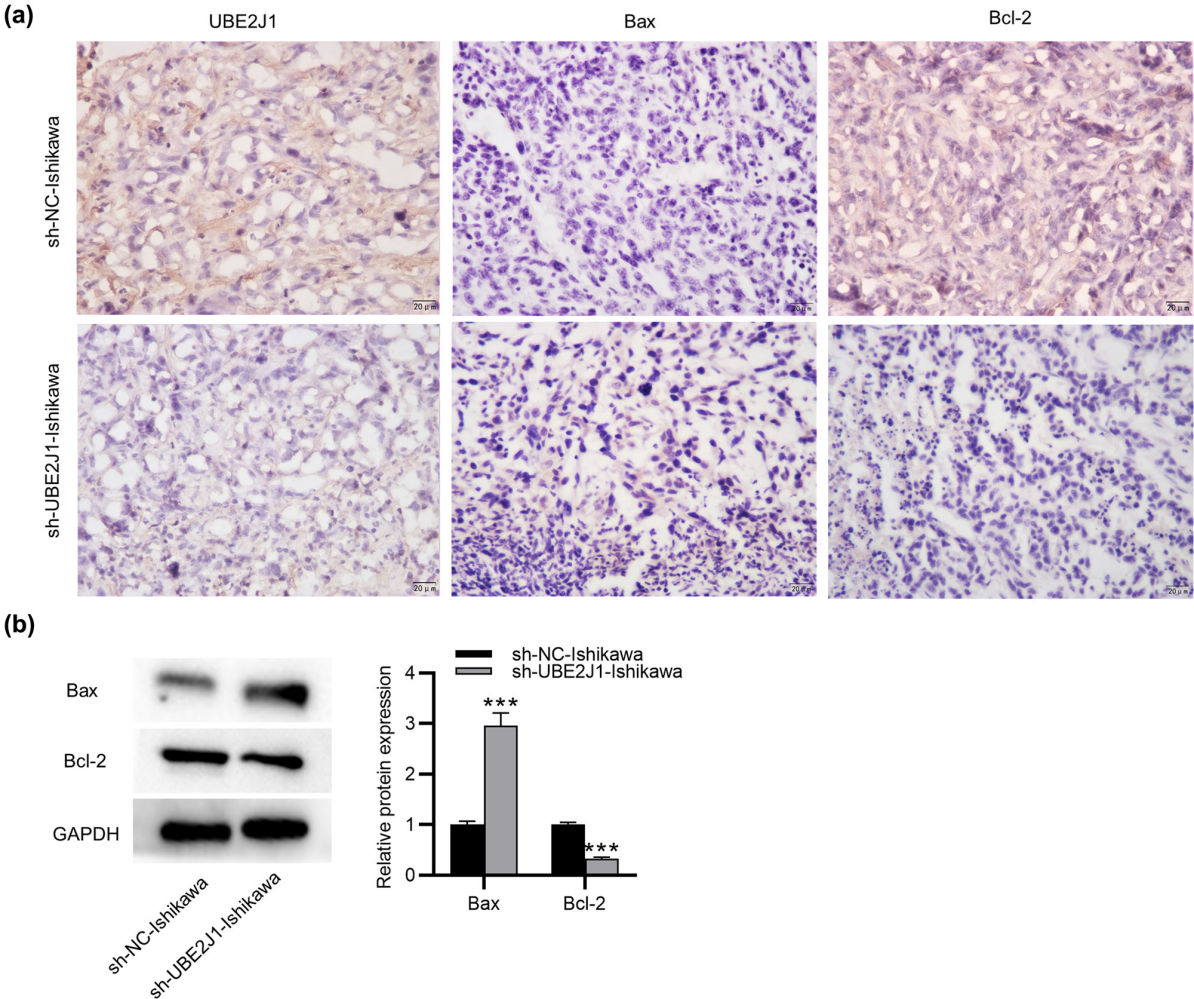
Knockdown of UBE2J1 induces cell apoptosis *in vivo*. (a) Expression levels of UBE2J1, Bax, and Bcl-2 in tumor tissues were detected by immunohistochemistry. (b) H-score for each protein. (c) Protein levels of Bax and Bcl-2 in tumor tissues were evaluated by western blot. Bar graphs were used to represent the data. ^***^ *p* < 0.001.

Given that UBE2J1 knockdown significantly facilitated EC cell apoptosis *in vitro*, we sought to evaluate the influence of UBE2J1 on cell apoptosis *in vivo* by using a mouse model of EC. The mice were subcutaneously injected with Ishikawa cells transfected with stable sh-UBE2J1 or sh-NC. Immunohistochemistry analysis demonstrated that the expression of UBE2J1 was lower in the sh-UBE2J1-Ishikawa group than in the sh-NC-Ishikawa group. The increased expression of Bax and the decreased expression of Bcl-2 were discovered in the sh-UBE2J1-Ishikawa group compared with the sh-NC-Ishikawa group (Figure 6a). In addition, western blot analysis indicated that Bax protein level was increased while Bcl-2 protein level was reduced in the sh-UBE2J1-Ishikawa group versus the sh-NC-Ishikawa group (Figure 6b). These results indicate that UBE2J1 depletion results in apoptosis induction *in vivo*.

### 3.7 Knockdown of UBE2J1 regulates the PI3K/AKT and MDM2/p53 signaling in the mouse model of EC

**Figure 7:**
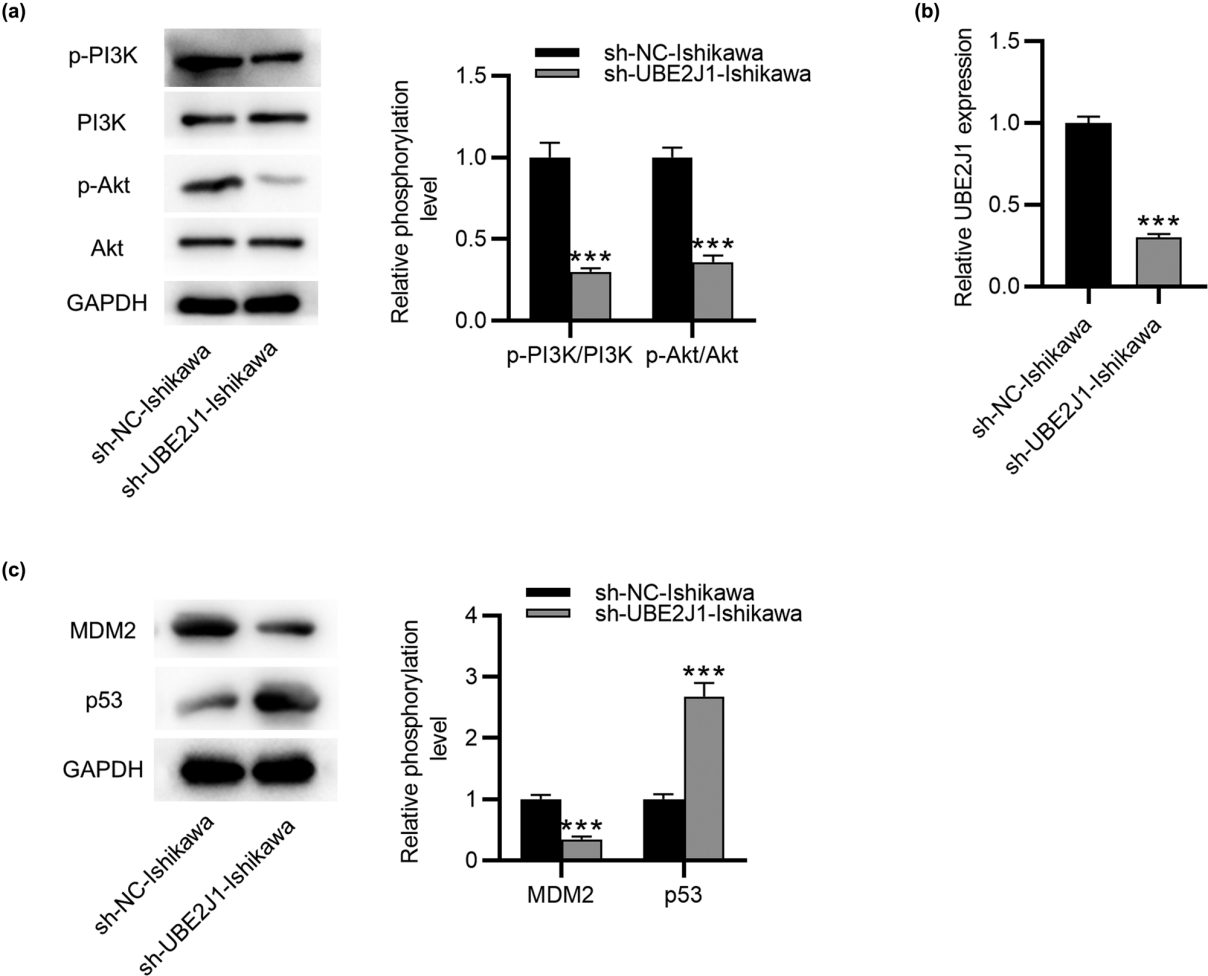
Knockdown of UBE2J1 regulates the PI3K/AKT and MDM2/p53 signaling *in vivo*. (a) Western blot analyses were applied to assess the protein levels of key molecules involved in the PI3K/AKT signaling pathway in tumor tissues. (b) Expression of UBE2J1 in tumor tissues was tested by RT-qPCR. (c) Protein levels of MDM2 and p53 in tumor tissues were examined by western blot. Bar graphs were used to represent the data. ^***^ *p* < 0.001.

Finally, the molecular mechanisms underlying the regulation of UBE2J1 on *in vivo* cell apoptosis were explored. Western blot analysis illustrated that the protein levels of p-PI3K and p-AKT were decreased in the sh-UBE2J1-Ishikawa group compared with the sh-NC-Ishikawa group (Figure 7a). Furthermore, lower expression of UBE2J1 and MDM2 and higher expression of p53 were found in the sh-UBE2J1-Ishikawa group than in the sh-NC-Ishikawa group (Figure 7b and c). Taken together, UBE2J1 knockdown suppressed the PI3K/AKT and activated the MDM2/p53 signaling in the mouse model of EC.

## 4 Discussion

Previous studies have revealed that UBE2J1 facilitates malignant phenotypes of many human cancers and is closely associated with poor overall survival and unfavorable prognosis of patients [7,8,16]. Importantly, downregulation of UBE2J1 was reported to repress EC cell growth, adhesion, migration, invasion as well as EMT process via mediating the PI3K/AKT signaling [9]. Based on this, our study demonstrated for the first time that UBE2J1 downregulation facilitated EC cell apoptosis, which might be achieved through regulating the PI3K/AKT and the MDM2/p53 signaling. Thus, we concluded from our study that UBE2J1 may be an oncogene in EC genesis and development.

The PI3K/AKT signaling is suggested to play an oncogenic role in multiple cancer cellular processes including cell proliferation, motion, and autophagy [17]. Moreover, emerging evidence has shown that UBE2J1 is involved in gene modulation in human cancers through different signaling pathways [9,18,19]. Herein, our study revealed that the expression of PI3K and AKT have positive correlations with that of UBE2J1. High expression PI3K and AKT contributes to poor prognosis in patients with EC. Since the previous study has demonstrated that UBE2J1 influenced EC cell phenotypes via modulating the PI3K/AKT pathway, the inhibition of UBE2J1 on EC cell apoptosis might also be mediated by through the PI3K/AKT axis. The subsequent western blot analyses in our study showed that UBE2J1 depletion markedly reduced the protein levels of p-PI3K and p-AKT in EC cells, suggesting that the PI3K/AKT pathway is implicated in the regulation of EC cell apoptosis by UBE2J1.

The p53 gene, as a tumor suppressor, plays a very important role in the progression of tumors [20]. P53 is usually inactivated during cancer development, which not only losses its suppressive tumor activities but often results in additional oncogenic functions, such as promoting cancer cell proliferation and metastasis, and inhibiting cell apoptosis [21,22]. The E3 ubiquitin protein ligase MDM2 was reported to negatively regulate p53, which can directly interact with p53 to suppress p53 transcriptional activity and translocation from the nucleus to the cytoplasm [23]. MDM2 is dysregulated in many cancers and impedes many activities that heavily depend on p53, such as cell apoptosis and cell-cycle arrest [24]. Numerous studies have demonstrated that the MDM2/p53 pathway plays a pivotal role in the development of a variety of tumors, including EC [25–27]. In addition, MDM2-mediated p53 ubiquitination and degradation can be modulated by the PI3K/AKT signaling pathway [28]. Herein, it was found that UBE2J1 positively modulated the expression of MDM2. UBE2J1 knockdown promoted p53 signaling by inhibiting MDM2 expression. Importantly, we found that the promotive effect of UBE2J1 knockdown on cell apoptosis in EC was reversed by MDM2 overexpression. We also analyzed the protein levels of cell apoptosis markers in cancer cells after the co-transfection of sh-UBE2J1 and pcDNA3.1/MDM2 to validate the interaction between UBE2J1 and MDM2. Typically, as a member of B cell leukemia 2 (BCL2) family, Bax serves as a regulator in various cell types and pro- or anti-apoptotic activities. Bax expression is modulated by p53 and was suggested to be implicated in p53-induced apoptosis [29–31]. Furthermore, *in vivo* animal experiments were also performed to verify the results obtained from *in vitro* assays. The results showed that UBE2J1 knockdown increased Bax protein level and decreased Bcl-2 protein level in mouse model of EC. The expression of p-PI3K, p-AKT, and MDM2 was elevated, while the expression of p53 was reduced in mice after downregulating UBE2J1. These analyses suggested that UBE2J1 can suppress the p53 pathway by upregulating MDM2 expression, thereby facilitating the malignancy of EC.

Our study has several strengths and limitations. This is the first study to investigate the role and molecular mechanism of UBE2J1 on cell apoptosis in EC. Second, *in vivo* animal experiments were performed to validate the findings of the *in vitro* assays. Third, we further confirmed that the PI3K/AKT pathway mediates the downstream MDM2/P53 signaling in EC development. However, there also exist some limitations. The upstream genes of UBE2J1 in mediating EC cell apoptosis have not been explored. Additionally, clinical data on EC cases were all obtained from public database, whose accuracy requires to be verified.

In conclusion, our study demonstrated for the first time that UBE2J1 suppresses cell apoptosis in EC by regulating the PI3K/AKT and MDM2/p53 signaling. Our findings provide new perspectives on the investigation of dysregulated genes in EC and suggest that the interaction among UBE2J1, the PI3K/AKT, and MDM2/p53 signaling pathway might be a possible therapeutic target for patients with EC.

## Supplementary Material

Supplementary Table

## Figures and Tables

**Table A1:**
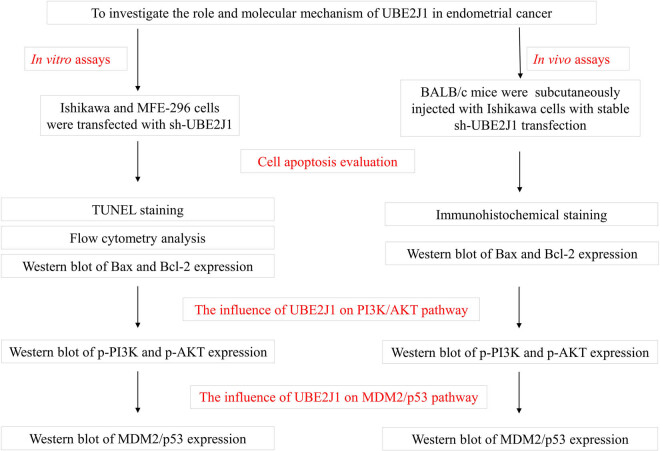
The detailed statistics for each assay.

## Appendix

Additional file 1 The flow chart of the main steps in our experiment.
